# Yearly changes in the composition of gut microbiota in the elderly, and the effect of lactobacilli intake on these changes

**DOI:** 10.1038/s41598-021-91917-6

**Published:** 2021-06-17

**Authors:** Ryuta Amamoto, Kazuhito Shimamoto, Sungjin Park, Hoshitaka Matsumoto, Kensuke Shimizu, Miyuki Katto, Hirokazu Tsuji, Satoshi Matsubara, Roy J. Shephard, Yukitoshi Aoyagi

**Affiliations:** 1 Food Research Department, Yakult Central Institute, Kunitachi, Tokyo Japan; 2 Exercise Sciences Research Group, Tokyo Metropolitan Institute of Gerontology, Itabashi, Tokyo Japan; 3 Microbiological Research Department, Yakult Central Institute, Kunitachi, Tokyo Japan; 4 Basic Research Department, Yakult Central Institute, Kunitachi, Tokyo Japan; 5 Faculty of Kinesiology and Physical Education, University of Toronto, Toronto, ON Canada

**Keywords:** Microbial communities, Microbiome, Microbiota

## Abstract

The onset and worsening of some diseases are related to the variation and instability of gut microbiota. However, studies examining the personal variation of gut microbiota in detail are limited. Here, we evaluated the yearly variation of individual gut microbiota in 218 Japanese subjects aged 66–91 years, using Jensen-Shannon distance (JSD) metrics. Approximately 9% of the subjects showed a substantial change, as their formerly predominant bacterial families were replaced over the year. These subjects consumed fermented milk products less frequently than their peers. The relationship between the intake frequencies of fermented milk products containing *Lacticaseibacillus paracasei* strain Shirota (LcS) and JSD values was also investigated. The intra-individual JSD of subjects ingesting LcS products ≥ 3 days/week over the past 10 years was statistically lower than the < 3 days/week group (*P* = 0.045). Focusing on subjects with substantial gut microbiota changes, only 1.7% of the subjects were included in the LcS intake ≥ 3 days/week group whereas 11.3% were found in the < 3 days/week group (*P* = 0.029). These results suggest that about one-tenth of the elderly Japanese could experience a substantial change in their gut microbiota during a 1-year period, and that the habitual intake of probiotics may stabilize their gut microbiota.

## Introduction

The human gut microbiota is a highly complex ecosystem, composed of a large number of bacteria (10^10^–10^11^cells/g feces) drawn from several hundreds of bacterial species^1^. The composition of the human gut microbiota is associated with certain types of disorders and the development of diseases, such as obesity^2^, diabetes^3,4^ and inflammatory bowel disease (IBD)^5,6^. In addition, some clinical trials have shown that a transplantation of fecal microbiota is highly effective in protecting against a recurrence of *Clostridioides difficile* infection^7,8^. Thus, the maintenance of favorable gut microbiota plays an important role in maintaining human health and in the prevention and treatment of many diseases.

The gut microbiota in human adults is generally recognized to remain stable in population-based studies. For example, most predominant bacterial species flourish in the human gut over periods of years^9,10^, and intra-individual (within-person) microbial variations are smaller than inter-individual (between-person) differences^10–13^. On the other hand, some studies have suggested that disturbance and instability in the gut microbiota are associated with risks to host health, such as exacerbation of human IBD^14^, neuro-inflammation and amyloidosis in a murine Alzheimer’s disease model^15,16^. Therefore, instability of gut microbiota may be considered as a potential risk to human health. However, studies examining details of the variation of gut microbiota within individuals are limited. Indeed, little is known about the incidence, causes and remedies for human microbial instability and the association between personal lifestyle and the temporal stability of gut microbiota.

Probiotics are defined by the World Health Organization (WHO)/Food and Agriculture Organization of the United Nations (FAO) as ‘Live microorganisms which confer a health benefit on the host when administered in adequate amounts’^17^. Typical examples are lactobacilli and bifidobacteria. Certain types/species of bacteria are also important to gastrointestinal health, and the processes involved appear to be facilitated by the regular ingestion of fermented milk products containing lactic acid bacteria. The ingestion of a specific probiotic bacterium, the *Lacticaseibacillus paracasei* strain Shirota (LcS), previously known as *Lactobacillus casei* strain Shirota^18^, is very popular both in Japan and world-wide; respective rates of consumption averaged about 8.9 and 41 million bottles per day in the year 2019^19^. LcS reaches the intestine alive^20–23^ and helps to ensure a good balance of gut microbiota^23–26^. Moreover, many other beneficial effects have been claimed for the regular ingestion of LcS, including improvement of bowel movements^23,25^, immunoregulation^27,28^ and protection against infection^24,29–31^. However, the impact of the regular consumption of the probiotics, including LcS products, on the stability of human gut microbiota remains unknown.

Over a 20-year period since 2000, we have been conducting an epidemiological study in the community of Nakanojo Town, Gunma Prefecture, Japan (the Nakanojo Study), assessing relationships between patterns of habitual physical activity and health in people aged 65 years and older^32–35^. Since 2014, we have also been collecting information on the subject’s frequency of the intake of fermented milk products, including LcS, and have previously reported the reduced risks of developing hypertension^36^ and infrequent bowel movements^37^ among elderly individuals who habitually take fermented milk products containing LcS.

Moreover, we started to analyze the subject’s fecal microbiota composition from 2015. These long-term collected data on the gut microbiota composition and the frequency of fermented product intake may provide an opportunity to explore the temporal variations of the gut microbiota in individual subjects and to identify any relationship between the consumption of probiotics and changes in microbiota composition.

The aim of this study was to assess the temporal variability of gut microbiota among elderly Japanese people over a 1-year period and to explore any possible associations between the intake frequency of fermented milk products containing LcS and the stability of the gut microbiota.

## Results

We selected 218 elderly Japanese people from the participants of the Nakanojo Study^32–35^, who provided fecal samples annually for 2 consecutive years or longer. The intra-individual yearly variations and the inter-individual differences in the composition of microbiota were assessed using Jensen-Shannon distance^38^ (JSD) and Bray–Curtis dissimilarity^39^ (BCD) metrics. Higher values of JSD and BCD indicate more different compositions of gut microbiota.

### The variations of gut microbiota within and between subjects

**Figure 1:**
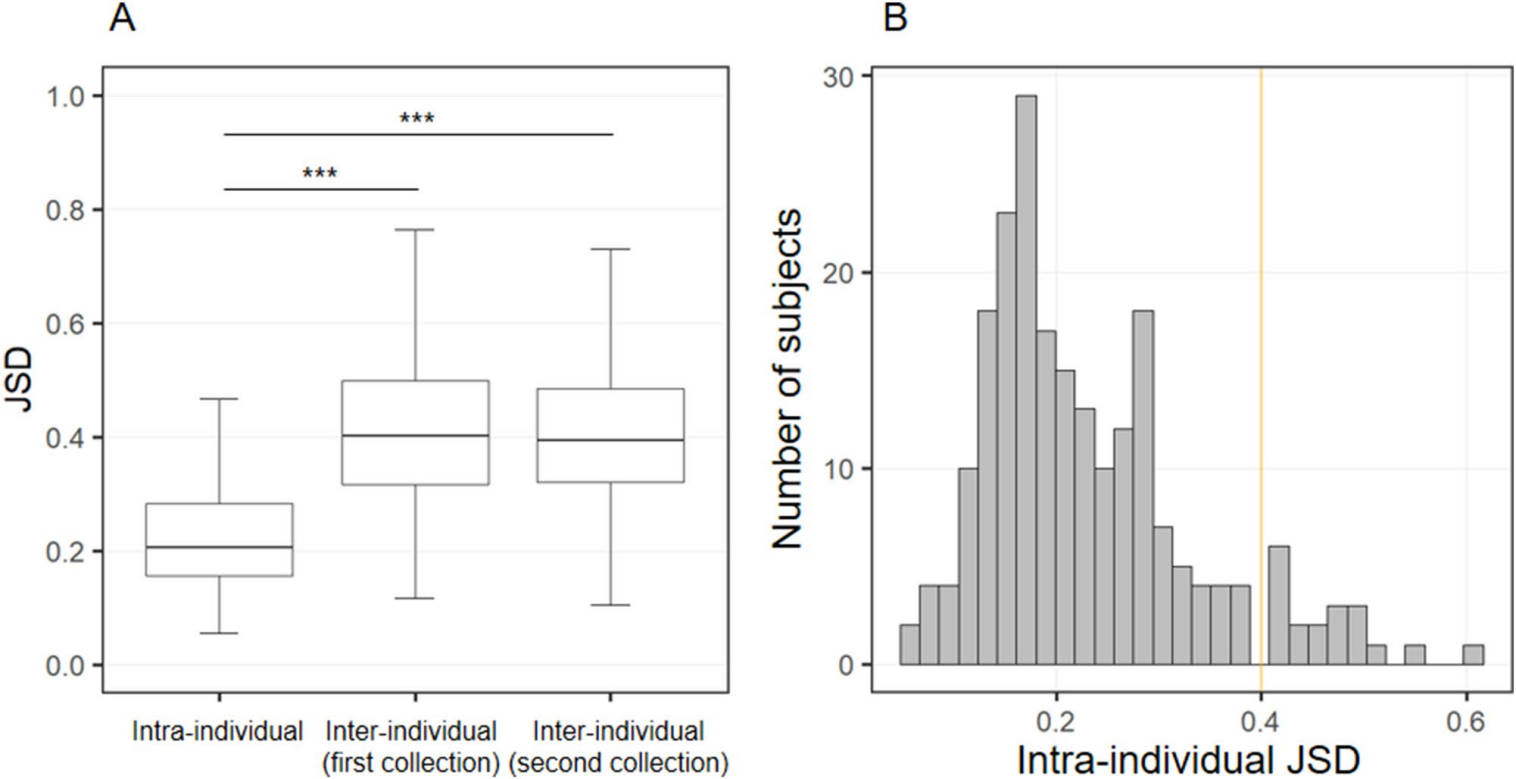
Intra-individual variations and inter-individual differences in gut microbial composition, quantified by Jensen-Shannon distance (JSD). (**A**) Comparison of JSD values between intra-individuals and inter-individuals by Steel’s tests. ***P < 0.001. (**B**) Distribution of intra-individual variations among the 218 subjects. The orange line indicates the median of the inter-individual JSDs (approximately 0.4).

Firstly, we compared the intra-individual temporal variations and inter-individual differences in the gut microbiota, and defined the subjects who experienced large temporal variations in their gut microbiota. The intra-individual JSD values (Supplementary Fig. S1A) of the gut microbial composition [median (minimum–maximum) of 0.205 (0.056–0.606)] were significantly smaller than the inter-individual differences [0.401 (0.117–0.763) over the first year and 0.395 (0.104–0.763) over the second year] (Fig. 1A). In terms of BCD metrics, similar results were observed (Supplementary Fig. S2). In this study, subjects whose intra-individual variability exceeded inter-individual differences in microbiota were defined as “subjects who experienced a substantial change in gut microbiota”. Thus, the threshold of intra-individual JSD was set at 0.4, which was equivalent to the median of inter-individual JSDs (0.4) and approximately twice the median of intra-individual JSDs (0.2).

### The compositional changes of gut microbiota in subjects with JSD ≥ 0.4

**Figure 2:**
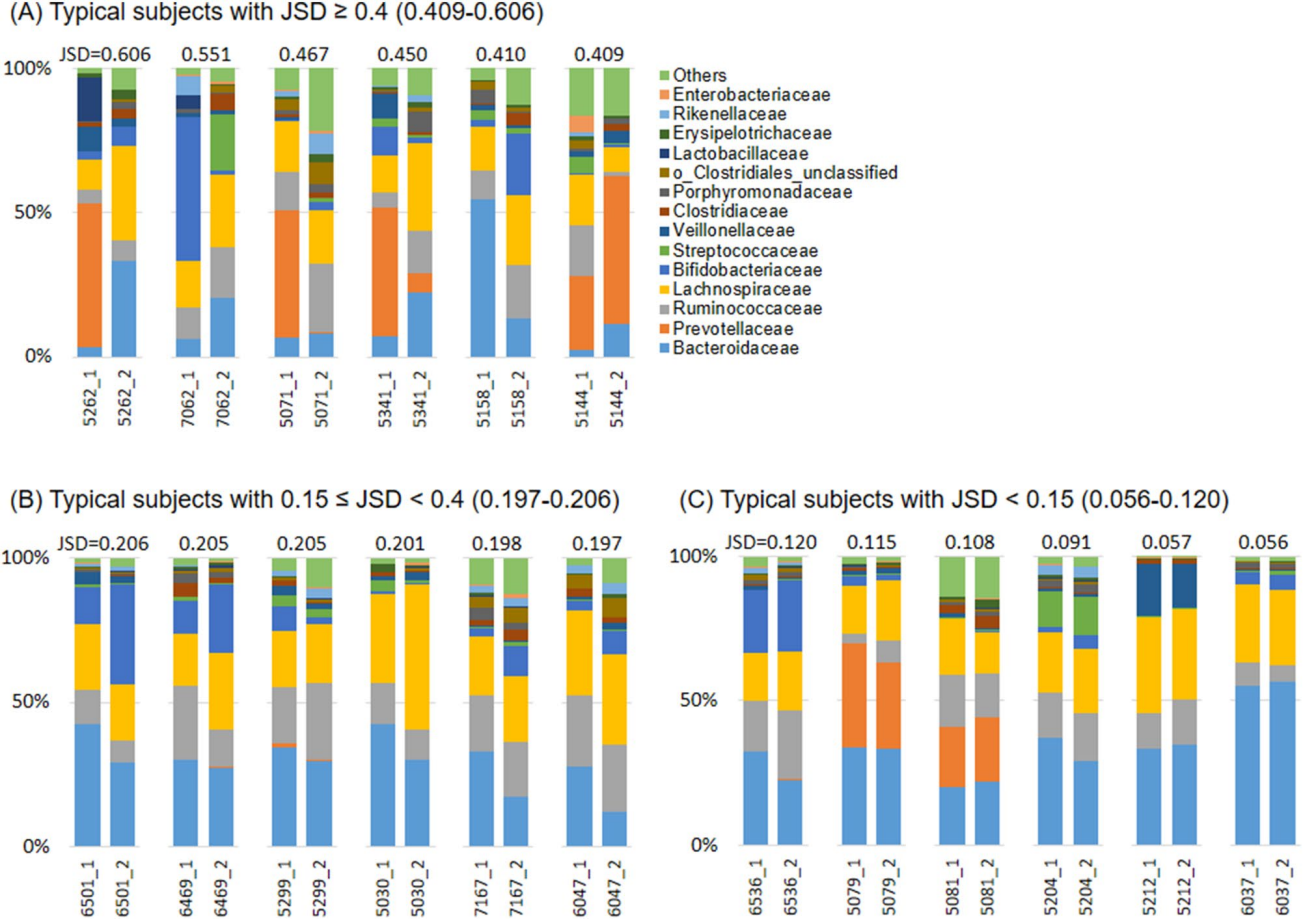
Changes of the predominant gut microbial composition at the family level between the first and second year. The subjects were classified into three groups by intra-individual Jensen-Shannon distance (JSD) range [(**A**) JSD ≥ 0.4 (large), (**B**) 0.15 ≤ JSD < 0.4 (moderate) or (**C**) JSD < 0.15 (small variations in their gut microbiota between the 2 years)]. The 6 respective representatives are shown.

Nineteen of 218 subjects (8.7%) had intra-individual JSD values higher than 0.4 (Fig. 1B), implying that they experienced a substantial change in the gut microbiota over a 1-year period. The detailed changes in the composition of the gut microbiota in annual fecal sample sets are presented in Fig. 2 and Supplementary Fig. S3. The 19 subjects with a JSD ≥ 0.4 showed that the noted compositional changes of microbiota were accompanied by the replacement of predominant bacteria at the family level over the 1-year period. In contrast, the remaining subjects with a JSD < 0.4 maintained the composition of the predominant bacterial community with moderate or little changes in their respective abundance.

Table 1 shows the background data for the 19 subjects with a JSD ≥ 0.4 and the other 199 subjects with lower JSD values. The percentages of current smokers and of those taking antibiotic medicines were significantly higher for the former than for the latter (15.8% vs 3.5% and 10.5% vs 0.5%, respectively). Also, the diastolic blood pressure was significantly lower in the former than in the latter (69.5 ± 13.3 mmHg vs 74.5 ± 9.6 mmHg), although there was no difference in the prevalence of hypertension between the groups (Supplementary Table S1). In addition, the intake frequencies of fermented milk products containing LcS were lower for the former than for the latter; the frequency for the past 10-years was about one-third for the former versus the latter. Supplementary Figure S4 presents the principal coordinate analysis plots of gut microbiota for subjects with JSDs < 0.4 and ≥ 0.4. Permutational multivariat analyses of variance revealed that there were no significant differences in the gut microbiota between the subjects of the two JSD groups at the first and second year.

**Table 1:** Characteristics of subjects with Jensen-Shannon distance (JSD) < 0.4 or ≥ 0.4.

	< 0.4		≥ 0.4	
	n	Mean ± SD	n	Mean ± SD
**Anthropometric parameters**				
Age (years)	199	75.5 ± 6.2	19	74.1 ± 6.4
Sex (male/female, %)	199	45.7/54.3	19	42.1/57.9
Height (m)	199	1.56 ± 0.09	19	1.55 ± 0.09
Body mass (kg)	199	55.7 ± 10.3	19	54.4 ± 9.7
Body mass index (kg/m^2^)	199	22.6 ± 3.1	19	22.7 ± 3.6
**Physical health measurements**				
Systolic blood pressure (mmHg)	195	127.3 ± 16.8	18	121.3 ± 19.8
Diastolic blood pressure (mmHg)	195	74.5 ± 9.6	18	69.5 ± 13.3*
Preferred walking speed (m/s)	179	1.40 ± 0.19	16	1.49 ± 0.21
Maximal walking speed (m/s)	178	1.99 ± 0.36	16	2.06 ± 0.29
Peak handgrip force (N)	185	271.1 ± 79.1	17	274.6 ± 82.9
Calcaneal osteosonic index (× 10^6^)	187	2.41 ± 0.35	18	2.35 ± 0.29
**Defecation status**				
Defecation frequency (days/week)	199	6.1 ± 1.5	19	6.1 ± 1.7
Bristol stool form scale score	199	4.0 ± 0.9	19	4.0 ± 1.0
**Habitual physical activity patterns**				
Step count (steps/day)	187	6983 ± 2988	18	8048 ± 3576
Duration of exercise > 3 metabolic equivalents (min/day)	187	16.5 ± 13.9	18	19.4 ± 16.9
**Current smoking and drinking habits**				
Smoker (number [%])	199	7 [3.5]	19	3 [15.8]*
Alcohol consumer (number [%])	199	76 [38.2]	19	10 [52.6]
**Medication states**				
Antibiotics (number [%])	199	1 [0.5]	19	2 [10.5]*
Gastric acid lowering medicines (number [%])	199	44 [22.1]	19	4 [21.1]
**Intake frequency of fermented milk products**				
LcS fermented milk products				
Past 1 month (days/week)	199	3.37 ± 2.91	19	1.84 ± 2.43*
Past 5 years (days/week)	199	2.35 ± 2.39	19	1.42 ± 2.27
Past 10 years (days/week)	199	1.82 ± 2.18	19	0.63 ± 1.67*
Overall fermented milk products				
Past 1 month (days/week)	199	5.29 ± 2.35	19	4.05 ± 2.63*
Past 5 years (days/week)	199	4.17 ± 2.53	19	3.47 ± 2.89
Past 10 years (days/week)	199	3.35 ± 2.55	19	2.47 ± 2.82
**Nutrient intakes**				
Energy (kcal/day)	188	2180 ± 604	18	2212 ± 543
Protein (g/day)	188	81.5 ± 24.7	18	84.4 ± 25.1
Lipid (g/day)	188	73.8 ± 28.0	18	72.9 ± 20.8
Carbohydrate (g/day)	188	283 ± 73	18	290 ± 69
Dietary fiber (g/day)	188	17.6 ± 5.3	18	19.2 ± 7.3
Saturated fatty acid (g/day)	188	22.6 ± 8.9	18	22.1 ± 7.1
Monounsaturated fatty acid (g/day)	188	24.5 ± 10.0	18	24.6 ± 6.6
Polyunsaturated fatty acid (g/day)	188	15.6 ± 5.9	18	16.4 ± 5.5
Cholesterol (mg/day)	188	371 ± 127	18	360 ± 111
Sodium (mg/day)	188	4737 ± 1795	18	5025 ± 2640
Potassium (mg/day)	188	3015 ± 963	18	3183 ± 1138
Calcium (mg/day)	188	803 ± 264	18	807 ± 309
Magnesium (mg/day)	188	316 ± 96	18	336 ± 119
Iron (mg/day)	188	9.91 ± 3.50	18	10.2 ± 3.7
Vitamin C (mg/day)	188	129 ± 51	18	135 ± 57
**Blood profiles**				
Triglyceride (mmol/l)	159	1.44 ± 0.75	13	1.57 ± 0.84
High-density lipoprotein cholesterol (mmol/l)	159	1.65 ± 0.55	13	1.52 ± 0.39
Low-density lipoprotein cholesterol (mmol/l)	159	3.17 ± 0.73	13	2.81 ± 0.58
Glycosylated hemoglobin A_1c_ (%)	159	5.64 ± 0.48	13	5.43 ± 0.17
Blood sugar (mmol/l)	159	6.15 ± 1.35	13	6.03 ± 1.07
Glutamic oxaloacetic transaminase (IU/l)	159	24.0 ± 6.0	13	27.6 ± 14.7
Glutamic pyruvic transaminase (IU/l)	159	19.3 ± 7.4	13	22.3 ± 15.2
γ-glutamyl transpeptidase (IU/l)	159	27.3 ± 26.4	13	27.7 ± 18.3
Albumin (g/l)	159	43.2 ± 3.1	13	41.8 ± 2.5
Creatinine (μmol/l)	159	74.9 ± 49.5	13	72.5 ± 25.2
Uric acid (μmol/l)	159	298 ± 74	13	291 ± 81
Estimated glomerular filtration rate (ml/min/1.73 m^2^)	159	62.7 ± 13.1	13	64.5 ± 14.7

Intergroup differences in the ratio of male/female, smoker/nonsmoker, alcohol consumer/non-alcohol consumer, and medicated/non-medicated were assessed by chi-square tests. Independent differences in each of the other variables between groups were assessed by analyses of covariance, after adjusting data on age for sex and the others for age and sex. *P < 0.05 versus JSD < 0.4.

Supplementary Table S2 summarizes the changes in medical history, medication status and lifestyle in subjects with a JSD ≥ 0.4 between the two fecal collections. Two subjects developed a new disease (ID 5002: ischemic enteritis, ID 6461: choledocholithiasis) during the year. Other 3 subjects took antibiotics or gastric acid lowering medication during the week preceding the second collection (ID 6202, 5084 and 5300). Eleven subjects with a JSD ≥ 0.4 had no changes in their medical history, medication status or lifestyle (ID 5262, 7062, 6086, 5398, 5378, 7137, 6442, 6636, 5400, 5158 and 5144). No consistent changes in physical activity patterns, smoking habits or alcohol consumption were observed between the two time points among the 19 subjects with a JSD ≥ 0.4.

### The persistence of temporal variations in individual gut microbiota

**Figure 3:**
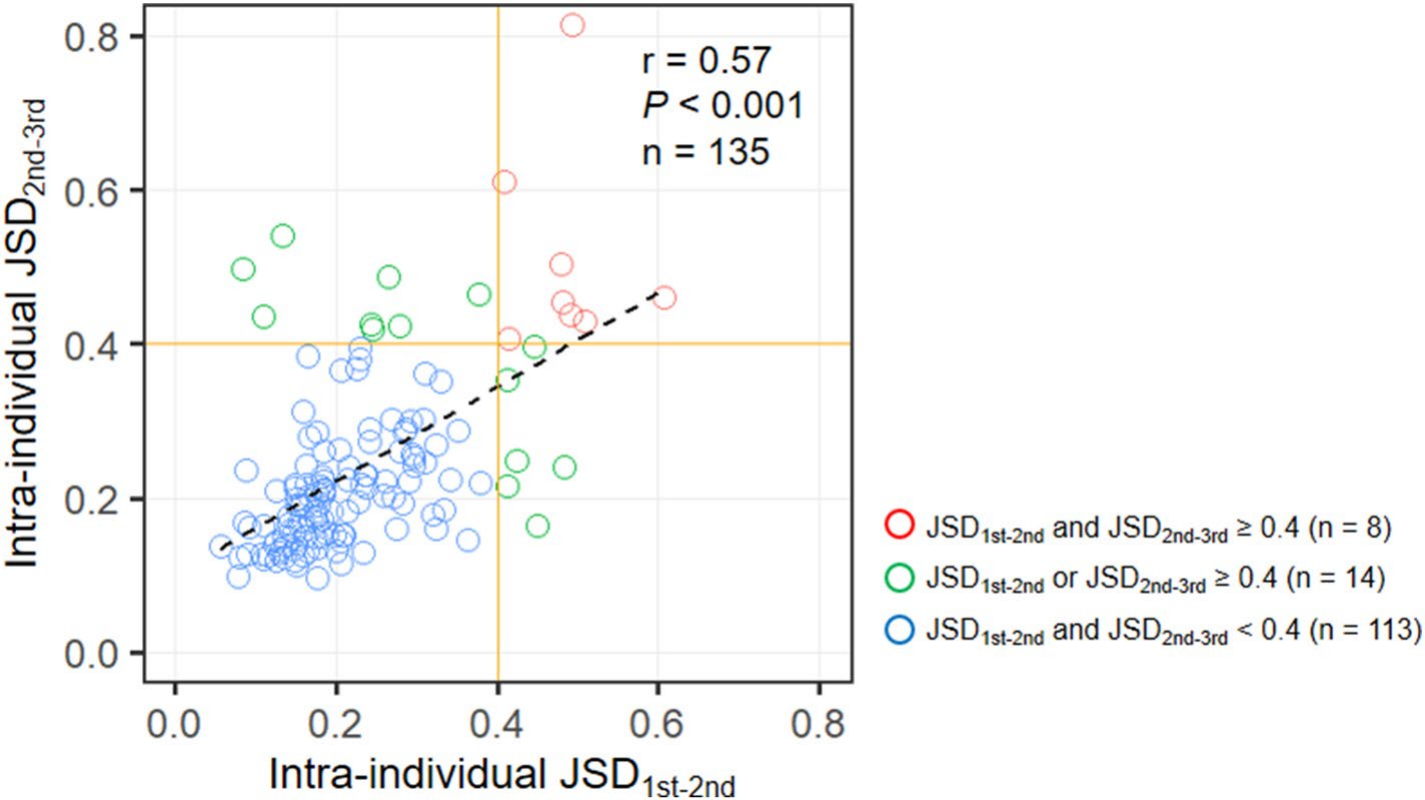
Association between intra-individual Jensen-Shannon distance from the first to the second (JSD_1st–2nd_) and from the second to the third (JSD_2nd–3rd_) fecal collections with respect to yearly changes in the gut microbiota. Spearman’s rank correlation analysis assessed the association between the JSDs during two periods.

To investigate the persistence of the temporal variability in gut microbiota, 135 subjects who provided their fecal samples for three consecutive years were selected from the 218 subjects validated in this study (Supplementary Fig. S1B). The correlation between the intra-individual JSDs of the first–second (JSD_1st–2nd_) and second–third fecal samples (JSD_2nd–3rd_) were analyzed. We noted that JSD_1st–2nd_ was strongly and significantly correlated with JSD_2nd–3rd_ (Fig. 3; r = 0.57, *P* < 0.001). For subjects who showed a substantial variation over the next year (JSD_2nd–3rd_ ≥ 0.4), 57.1% (8/14) were found in the subjects with a JSD_1st–2nd_ ≥ 0.4 compared to only 6.6% (8/121) with a JSD_1st–2nd_ < 0.4; a Fisher’s exact test showed the significant difference in the prevalence of JSD_2nd–3rd_ ≥ 0.4 between these two groups (*P* < 0.001). In 83.7% of the subjects (113/135), both JSD_1st–2nd_ and JSD_2nd–3rd_ showed < 0.4, implying that there had been no substantial change in their gut microbiota over the 2 years. On the other hand, 10.4% (14/135) of the subjects experienced a substantial change in their gut microbiota once within the 2 year, with values of 0.4 or higher in either JSD_1st–2nd_ or JSD_2nd–3rd_. Furthermore, 5.9% (8/135) showed continuous gut microbiota changes over the 2 years, with values of 0.4 or higher in both JSD_1st–2nd_ and JSD_2nd–3rd_. Supplementary Figure S5 shows the changes in the composition of gut microbiota for these 8 subjects in 3 fecal collections. In 3 of the 8 subjects, JSD_1st–3rd_ was also 0.4 or higher, suggesting that the composition of microbiota was remarkably different in each fecal collection. In the remaining 5 subjects, JSD_1st–3rd_ was lower than 0.4; thus, the composition of their gut microbiota returned to the initial state in the 3rd sample collection (Supplementary Fig. S5).

### Relationships between the intake frequency of fermented milk products and temporal variations of gut microbiota

**Figure 4:**
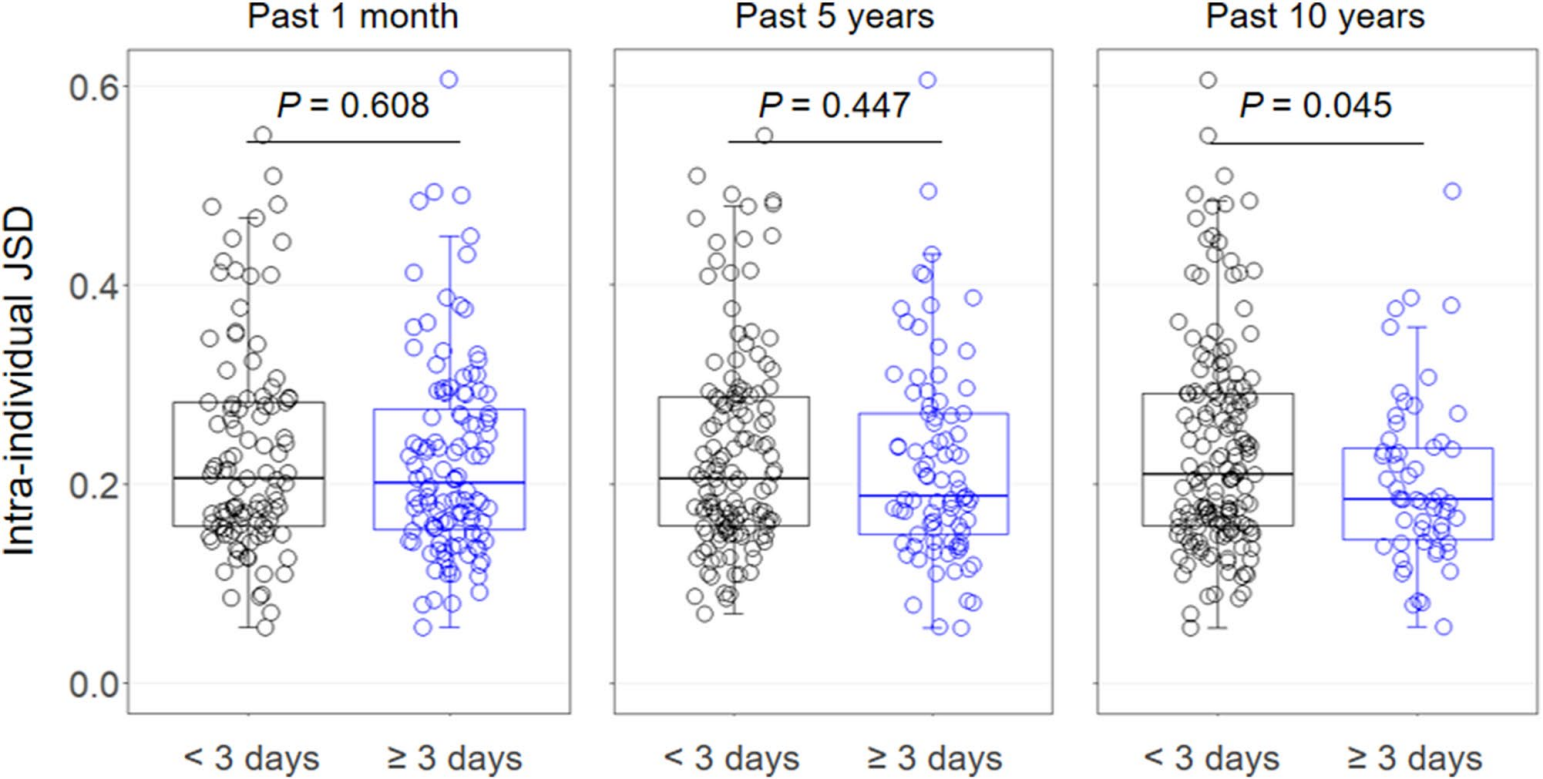
Intra-individual Jensen-Shannon distance (JSD) in subjects consuming fermented milk products containing *Lacticaseibacillus paracasei* strain Shirota (LcS) < 3 or ≥ 3 days/week during the past 1-month, 5-year and 10-year periods. Independent differences in the JSD between groups were assessed by analyses of covariance, after adjusting data for age, sex, body mass index, smoking status, and alcohol intake.

We investigated the relationship between the intake frequencies of fermented milk products (Supplemental Table S3) and the temporal variation of gut microbiota over a 1-year period in the 218 subjects mentioned above (Fig. 1B). Among subjects ingesting LcS products ≥ 3 days/week over a 10-year period, the intra-individual JSD of gut microbial composition was statistically lower than that of the < 3 days/week group [median (minimum–maximum) of 0.185 (0.057–0.494) and 0.211 (0.056–0.606), respectively, *P* = 0.045] (Fig. 4). In addition, the percentages of the subjects whose intra-JSDs showed ≥ 0.4 were 11.3% (18/160) in the < 3 days/week group but only 1.7% (1/58) in the ≥ 3 days/week group; a Fisher’s exact test showed that this 9% difference between the two groups was statistically significant (*P* = 0.029). With respect to intake over a 1-month and a 5-year period, the intra-individual JSD and the incidence of major variation of gut microbiota in the ≥ 3 days/week group tended to be similarly lower than those for the < 3 days/week group, although insignificantly so (Fig. 4). In terms of overall fermented milk products, the corresponding differences in the intra-individual JSD were not statistically significant (Supplementary Fig. S6). Similar results were observed by the BCD metrics (Supplementary Fig. S7).

Table 2 shows the background data of subjects ingesting LcS products < 3 and ≥ 3 days/week over a 10-year period. Moderate alcohol consumers showed 45.6% (73/160) in the < 3 days/week group and 22.4% (13/58) in the ≥ 3 days/week group, with this inter-group difference being statistically significant (*P* < 0.05). On the other hand, no significant differences were detected in the taking of antibiotics or gastric acid lowering medicines between < 3 and ≥ 3 days/week groups. Furthermore, even after adjusting for the intake of antibiotics or gastric acid lowering medicines, the values of intra-individual JSD remained lower in subjects ingesting LcS products ≥ 3 rather than < 3 days/week (*P* = 0.055 or 0.048, respectively; data not shown). There were no statistically significant differences between the two groups with respect to any of the other variables examined, except the intake of vitamin C. In terms of the other intake periods and intake of overall fermented milk products, similar results were observed (data not shown).

**Table 2:** Characteristics of subjects consuming fermented milk products containing *Lacticaseibacillus paracasei* strain Shirota (LcS) < 3 or ≥ 3 days per week over the past 10-year period.

	< 3 days/week		≥ 3 days/week	
	n	Mean ± SD	n	Mean ± SD
**Anthropometric parameters**				
Age (years)	160	75.1 ± 6.3	58	76.0 ± 6.0
Sex (male/female, %)	160	48.8/51.3	58	36.2/63.8
Height (m)	160	1.57 ± 0.09	58	1.55 ± 0.09
Body mass (kg)	160	55.9 ± 10.6	58	54.5 ± 9.1
Body mass index (kg/m^2^)	160	22.7 ± 3.3	58	22.5 ± 2.7
**Physical health measurements**				
Systolic blood pressure (mmHg)	157	125.7 ± 16.3	56	130.1 ± 19.0
Diastolic blood pressure (mmHg)	157	73.9 ± 10.1	56	74.6 ± 9.8
Preferred walking speed (m/s)	145	1.40 ± 0.20	50	1.41 ± 0.15
Maximal walking speed (m/s)	144	2.00 ± 0.37	50	1.98 ± 0.30
Peak handgrip force (N)	149	274.5 ± 78.5	53	262.8 ± 81.2
Calcaneal osteosonic index (× 10^6^)	151	2.42 ± 0.36	54	2.38 ± 0.30
**Defecation status**				
Defecation frequency (days/week)	160	6.1 ± 1.5	58	5.9 ± 1.7
Bristol stool form scale score	160	4.0 ± 0.9	58	3.9 ± 0.9
**Habitual physical activity patterns**				
Step count (steps/day)	151	7085 ± 3041	54	7053 ± 3102
Duration of exercise > 3 metabolic equivalents (min/day)	151	17.2 ± 14.2	54	15.6 ± 14.1
**Current smoking and drinking habits**				
Smoker (number [%])	160	9 [5.6]	58	1 [1.7]
Alcohol consumer (number [%])	160	73 [45.6]	58	13 [22.4]**
**Medication states**				
Antibiotics (number [%])	160	2 [1.3]	58	1 [1.7]
Gastric acid lowering medicines (number [%])	160	31 [19.4]	58	17 [29.3]
**Nutrient intakes**				
Energy (kcal/day)	152	2166 ± 624	54	2230 ± 520
Protein (g/day)	152	81.7 ± 26.2	54	82.0 ± 20.1
Lipid (g/day)	152	73.3 ± 28.1	54	74.8 ± 25.6
Carbohydrate (g/day)	152	279 ± 75	54	297 ± 65
Dietary fiber (g/day)	152	17.5 ± 5.8	54	18.5 ± 4.3
Saturated fatty acid (g/day)	152	22.6 ± 9.2	54	22.6 ± 7.6
Monounsaturated fatty acid (g/day)	152	24.6 ± 10.2	54	24.0 ± 8.2
Polyunsaturated fatty acid (g/day)	152	15.7 ± 6.1	54	15.4 ± 5.1
Cholesterol (mg/day)	152	373 ± 131	54	359 ± 109
Sodium (mg/day)	152	4753 ± 2053	54	4788 ± 1272
Potassium (mg/day)	152	3004 ± 1035	54	3100 ± 800
Calcium (mg/day)	152	788 ± 273	54	847 ± 249
Magnesium (mg/day)	152	317 ± 105	54	321 ± 77
Iron (mg/day)	152	9.70 ± 3.47	54	10.6 ± 3.58
Vitamin C (mg/day)	152	125 ± 52	54	142 ± 49*
**Blood profiles**				
Triglyceride (mmol/l)	124	1.47 ± 0.75	48	1.40 ± 0.76
High-density lipoprotein cholesterol (mmol/l)	124	1.63 ± 0.58	48	1.68 ± 0.45
Low-density lipoprotein cholesterol (mmol/l)	124	3.10 ± 0.75	48	3.27 ± 0.65
Glycosylated hemoglobin A_1c_ (%)	124	5.65 ± 0.50	48	5.56 ± 0.34
Blood sugar (mmol/l)	124	6.22 ± 1.44	48	5.93 ± 0.98
Glutamic oxaloacetic transaminase (IU/l)	124	23.9 ± 7.3	48	25.2 ± 6.2
Glutamic pyruvic transaminase (IU/l)	124	19.7 ± 8.5	48	19.0 ± 7.2
γ-Glutamyl transpeptidase (IU/l)	124	29.3 ± 29.0	48	22.5 ± 13.7
Albumin (g/l)	124	42.9 ± 3.0	48	43.6 ± 3.2
Creatinine (μmol/l)	124	76.6 ± 55.7	48	69.8 ± 15.5
Uric acid (μmol/l)	124	300 ± 76	48	293 ± 71
Estimated glomerular filtration rate (ml/min/1.73 m^2^)	124	63.3 ± 14.3	48	61.7 ± 9.6

Intergroup differences in the ratio of male/female, smoker/nonsmoker, alcohol consumer/non-alcohol consumer, and medicated/non-medicated were assessed by chi-square tests. Independent differences in each of the other variables between groups were assessed by analyses of covariance, after adjusting data on age for sex and the others for age and sex. *P < 0.05, **P < 0.01 versus < 3 days/week.

Data on 1-month and 5-year periods are not shown, mainly because of their similarities to those on a 10-year period.

**Figure 5:**
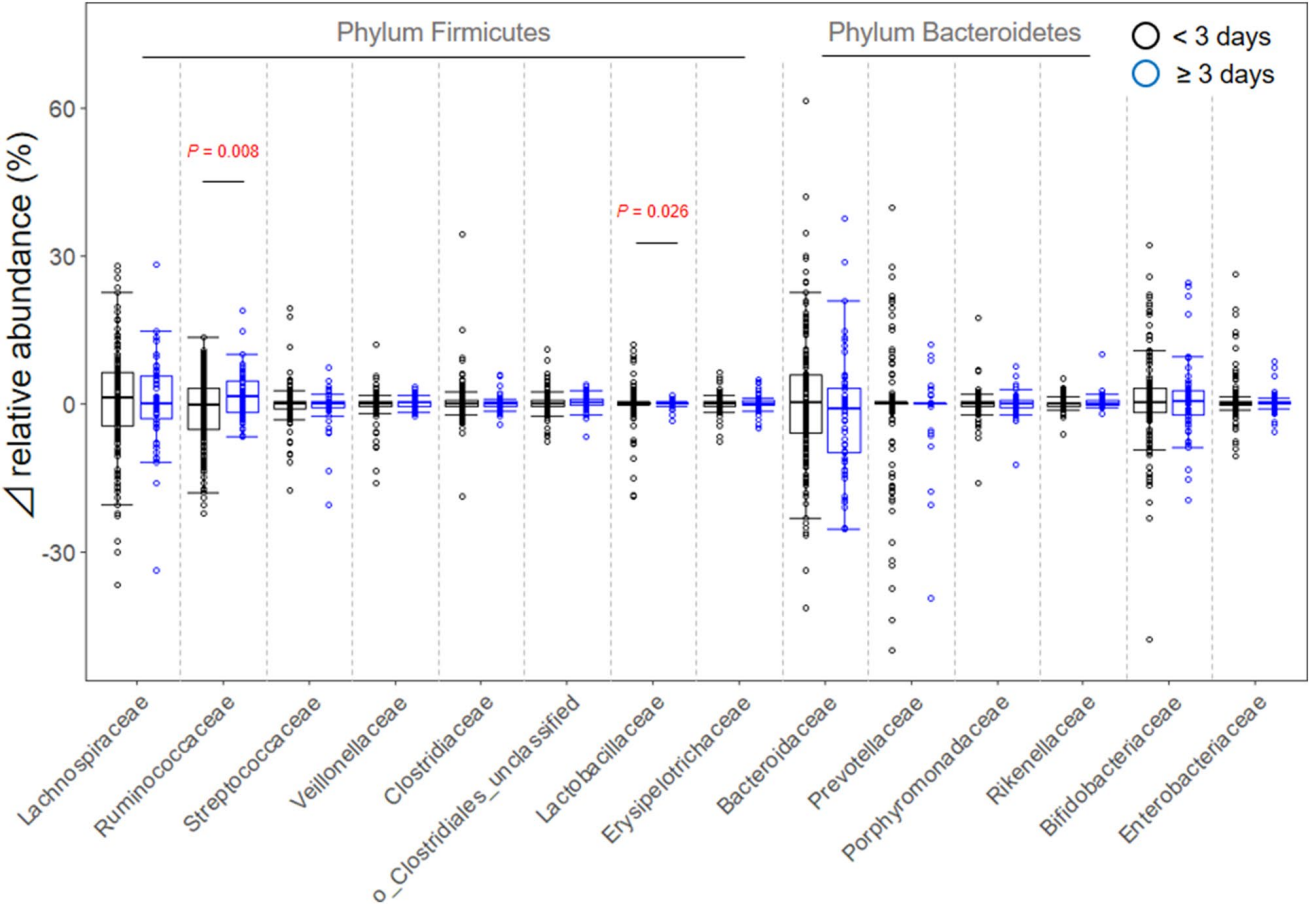
Changes in the relative abundance of each predominant bacteria during a year in subjects consuming fermented milk products containing *Lacticaseibacillus paracasei* strain Shirota (LcS) < 3 or ≥ 3 days/week over the past 10-year period. Independent differences between groups in terms of dispersions of changes in the abundance were assessed by Levene’s tests. *P* values with < 0.05 are shown.

Figure 5 shows net changes in the abundance of each bacterial family during a year. In all bacterial families, the individual changes of relative abundance across the two time points were distributed in both positive (increasing) and negative (decreasing) directions, with their medians showing approximately 0% in both groups of LcS intake < 3 and ≥ 3 days/week. On the other hand, comparing dispersions of variations in each bacterial abundance between the two groups by Levene’s tests showed that Ruminococcaceae and Lactobacillaceae in the ≥ 3 days/week group were significantly lower than those in the < 3 days/week group (Fig. 5, *P* = 0.008 and 0.026, respectively).

## Discussion

This epidemiological study showed that the gut microbiota of the general Japanese elderly people is comprehensively stable (Fig. 1), supporting other population-based observations^9–13^. However, approximately 9% (19/218) of the subjects showed considerable changes in the composition of the gut microbiota over a year, with a replacement of the predominant bacterial families. Moreover, a strong positive correlation was observed between JSD_1st–2nd_ and JSD_2nd–3rd_ (Fig. 3), among the subjects who provided feces for 3 consecutive years. These results suggest that the stability of the gut microbiota composition in the elderly persists, and those who experience major changes in their gut microbiota are likely to have such changes subsequently. No common direction or trend in the changes of gut microbiota composition was observed (Figs. 2, and 5).

Among the 19 subjects who experienced a substantial change in the gut microbiota (JSD ≥ 0.4 group), 2 subjects developed new pathologies (ischemic enteritis and choledocholithiasis) over the year, and 3 other subjects took antibiotics or gastric secretion inhibitors just before the second fecal collection (Supplementary Table S2). Moreover, the 3-year investigation showed that continuous and significant changes in the gut microbiota were observed annually in 3 subjects (ID 5002, 5084, 5144; Supplementary Fig. S5). ID 5002 was the only subject who developed ischemic enteritis between the first and second year, and ID 5084 was found to be taking antibiotics. On the other hand, there were subjects whose gut microbiota changed significantly, but then returned to the state before the change (ID 6202, 6636, 5262, 5398, 6086; Supplementary Fig. S5). Among these subjects, one subject (ID 6202) was taking antibiotics at the second fecal collection, and unlike ID 5084 above, the changes in microbiota were transient. We attempted to determine the cause of the changes in the gut microbiota based on the personal characteristics collected through this study, but did not lead to clarification of the responsible factors. These results suggest that there might be “individual characteristics” that cannot be explained by the use of antibiotics, and changes in disease onset, physical condition and/or lifestyle habits. Relationships between the onset of disease and/or long-term effects of antibiotic use and the ongoing changes in the gut microbiota remain unknown. Because the number of subjects whose gut microbial composition changed was small in this study, larger-sample studies are needed to clarify the factors that influence continuous or transient changes in gut microbiota.

We have also done a comparison on the baseline characteristics between subjects who changed in their gut microbiota (JSD ≥ 0.4 group) and those who did not (JSD < 0.4 group). The percentages of antibiotics users and current smokers were significantly greater in the JSD ≥ 0.4 group compared to their peers (Table 1). However, antibiotic takers and current smokers were only 2 and 3 of the 19 subjects, respectively, suggesting limited effects on the substantial changes of gut microbiota.

Diastolic blood pressure was significantly lower in the JSD ≥ 0.4 group than that in the JSD < 0.4 group (Table 1), suggesting that blood pressure may be affected by microbiota changes, although both mean values were within an optimal range of diastolic pressure. On the other hand, the cause-and-effect relationship was unidentified in this study. Since previous studies have reported that gut microbiota may affect blood pressure^40,41^, it is possible that changes in the microbiota may affect the blood pressure. Future studies will be necessary to clarify the causal relationship and its clinical importance through prospective and interventional studies.

Many other factors such as the intake of dietary fiber and the frequency of defecation also have an influence on gut microbiota^42,43^. Although the nutrient intake and stool frequency were similar for two groups of subjects with different JSDs, the intake frequencies of products containing lactic acid bacteria in subjects with a JSD ≥ 0.4 were less than those of their peers (Table 1).

Lactic acid bacteria have been used in the production of fermented food and patronized in all over the world since ancient times, and LcS is recognized as a representative probiotic strain. The present investigation confirmed statistically significant differences in the intra-individual variation of gut microbiota over a year between subjects taking LcS fermented milk products < 3 and ≥ 3 days/week for the past 10 years. The incidence of “substantial variation of gut microbiota” in the latter subjects was less than one-fifth of that in the former subjects. Likewise, lower dispersions of variations in the bacterial abundance of Ruminococcaceae and Lactobacillaceae over a year were seen in subjects ingesting LcS products ≥ 3 rather than < 3 days/week. Therefore, it appears that habitual intake of fermented milk products containing LcS is associated with a more stable gut microbiota composition, mainly by suppressing the variations of these bacterial families.

LcS has been shown to have a high tolerance to gastric and bile acids^44^ and to reach the intestine alive^20–23^, suggesting that the production of organic acids such as lactic acid is increased^45^, lowering pH in the intestine. LcS is also known to provide a good balance of gut microbiota both by increasing the abundance of beneficial bacteria such as bifidobacteria and lactobacilli and by decreasing the abundance of harmful bacteria^23–26^. Together, these improvements in gut microbiota and environments by LcS may contribute directly to the stability of the composition of gut microbiota, leading to the stabilization of gut microbiota among subjects taking LcS-containing fermented milk products ≥ 3 days/week.

Furthermore, LcS is known to have a preventive effect of infection^24,29–31^, which may reduce the frequency of drug use and consequently stabilize gut microbiota. However, no differences were detected in the taking of antibiotics or gastric acid lowering medicines between < 3 and ≥ 3 days/week groups, and the values of intra-individual JSD remained lower in the ≥ 3 days/week group after adjusting for the intake of antibiotics or gastric acid lowering medicines. Thus, the intake of antibiotics or gastric acid lowering medicines does not appear to have great effect on the relationship between the intake frequency of lactic acid bacteria and the stability of microbiota.

Other factors such as stress suppression may be involved in the stabilization of gut microbiota by LcS, as LcS has been reported to suppress naturalistic stresses^46–48^. Moreover, LcS has also been reported to have immunoregulatory effects, including the enhancement of natural killer cell activity^49–51^ and the maintenance of salivary Immunoglobulin A (IgA) level^28^. Recent reports have suggested that gut microbiota are regulated by host IgA^52–54^, so that the effect of LcS on humoral immunity of the host may have contributed to the stabilization of gut microbiota. Further studies are needed to clarify the mechanism of higher stability among subjects who habitually take fermented milk products containing LcS.

There are some limitations to the current investigation. The design was partly retrospective and observational rather than prospective and experimental, so that causation cannot be inferred. The estimated frequency of LcS intake was based on questionnaires completed regularly and repeatedly by the subjects, using a standardized methodology^36,37^. Furthermore, the relative abundances of the gut microbiota were determined using the standardized methodology of the 16S ribosomal RNA gene amplicon analysis^37,47^. These facts strengthen the practical significance of the observed relationship between the frequency of LcS-containing product consumption and intra-individual variations in gut microbiota. On the other hand, individuals ingesting fermented milk supplements ≥ 3 days/week might differ from their peers in terms of a greater overall interest in a healthy lifestyle, including other facets of behavior that could limit instability in their gut microbiota. Among the lifestyle covariates we examined, and with the exception of less alcohol consumption and more vitamin C consumption in those ingesting fermented milk ≥ 3 days/week, the two groups appeared to be well matched. Moreover, we covaried the major important lifestyle determinants of gut microbiota (age, sex, body mass index, cigarette smoking and alcohol consumption), although the statistical adjustment for these factors may have been incomplete. Although most people drink up the entire bottle of LcS, the amount and frequency of fermented milk intake are not necessarily closely related. Also, the variations of gut microbiota are linked to many other aspects of food intake, such as the content of dietary fiber and cereals, and the response to a frequent intake of LcS-containing fermented milk might diverge in a population whose diet differed substantially from the rice-based nutrition of the elderly in rural Japan. In addition, we could not consider the impact of uncollected data such as stress, long-term status of medication and changes in family structure on the intra-individual stability of gut microbiota. Therefore, it is necessary to replicate this study in populations of various ages and consuming various diets. Moreover, relationships between the instability of gut microbiota and potential risks to human health are still unresolved, and prospective studies are required to examine the clinical significance of the stability of gut microbiota and the effectiveness of continuous intake of LcS fermented milk products.

In conclusion, our study suggests that about one-tenth of elderly Japanese could experience a substantial compositional change in the predominant gut microbiota during a year. Also, those who experienced major changes in their gut microbiota were more likely to have subsequent microbiota changes. These results suggest that the individual stability of gut microbiota is continuously maintained and is regulated by lifestyle habits. Moreover, this study has shown for the first time that the habitual intake of probiotics, particularly LcS, may stabilize the elderly’s gut microbiota. Elucidating the mechanism of gut microbiota stabilization through the intake of probiotics and how this gut microbiota stabilization affects the health of the elderly may provide hints for the construction of a healthy society with longevity.

## Methods

### Subjects

The subjects were self-supporting and independent Japanese volunteers aged 65 years or older who had been recruited to the Nakanojo Study^32–35^. Criteria of recruitment included attendance at an annual medical examination, functional independence, and the absence of chronic or progressive conditions that could limit physical activity or have a major effect on the individual’s perceived quality of life (e.g., cancer, arthritic diseases, Parkinson’s disease, Alzheimer’s disease, multiple sclerosis, amyotrophic lateral sclerosis, and dementia). In the present study, we selected 218 subjects (99 males and 119 females) aged 66–91 years who provided fecal samples once a year for at least 2 consecutive years (Fig. S1).

### Collection and treatment of fecal samples

Fecal samples were collected and treated as described previously^37^. In brief, subjects were given sterile feces tubes (Sarstedt AG & Co., Nümbrecht, Nordrhein-Westfalen, Germany) containing 2 ml of a ribonucleic acid (RNA) stabilization solution (RNA*later*; Thermo Fisher Scientific Inc., Waltham, MA, United States) for the analyses of gut microbiota. Using the collection spoon attached to the tube cap, each subject placed a spoonful of fecal sample (approximately 500 mg) into each collection tube immediately after defecating. Samples were stored at room temperature and sent to the Yakult Central Institute within a couple of days. After arrival at the Institute, samples were weighed to calculate the fecal volume (average weight 475 ± 347 mg), suspended in nine volumes of RNA*later*, and then homogenized. Two hundred μl of the fecal homogenate (equivalent to 20 mg of feces) was added to 1 ml of sterilized phosphate-buffered saline and then centrifuged at 10,000×*g* for 5 min. The supernatant (1 ml) was discarded, and the resulting fecal homogenate was stored at − 80 °C until deoxyribonucleic acid (DNA) extraction.

### Assay of gut microbiota

The relative abundances of the fecal bacterial families were determined by amplicon sequencing analysis targeting the 16S ribosomal RNA (rRNA) gene, as described previously^37,47^, using the open-source Quantitative Insights Into Microbial Ecology (QIIME) software^55^. Briefly, DNA was extracted from fecal samples^56^, and the V1–V2 region of the 16S rRNA gene of gut microbiota was amplified using the forward primer 27Fmod2-MiSeqV2 and the reverse primer 338RMiSeqV2-001^44^. The amplicons were purified using an Agencourt AMPure XP (Beckman Coulter K. K., Koto, Tokyo, Japan), quantified using a Quant-iT PicoGreen dsDNA (Thermo Fisher Scientific Inc., Waltham, MA, United States), and sequenced using a MiSeq sequencing system (Illumina K. K., Minato, Tokyo, Japan).

### Evaluation of gut microbiota variation

Differences in the relative abundance of the bacterial family composition between and within subjects were evaluated by calculating the JSD metrics^38^, which are generally used in enterotypes classification of the human gut microbiome^57^. The difference *JSD* (*a*, *b*) between samples *a* and *b* is defined as

$$JSD (a, b)=\sqrt{JSd ({P}_{a},{P}_{b})}$$

where *P*_*a*_ and *P*_*b*_ are the abundance distributions of samples *a* and *b* and *JSd* (*x*_,_ *y*) is the *Jensen-Shannon divergence* between two probability distributions x and y defined as

$$JSd=\frac{1}{2}KLD(x,m)+\frac{1}{2}KLD(y,m)$$

where $m=\frac{x+y}{2}$ and *KLD* (*x*, *y*) is the Kullback–Leibler divergence between *x* and *y* defined as

$$KLD(x,y)=\sum_{i}{x}_{i}\mathrm{log}\frac{{x}_{i}}{{y}_{i}}$$

We added a pseudocount of 0.0000001 to the abundance distributions and renormalized them to avoid zero in the numerator and/or denominator of equation. The differences in the composition of gut microbiota were also evaluated by calculating a BCD metrics^39^ using “vegdist” function in a “vegan” package of R software (version 3.3) (https://cran.r-project.org/)^58^. With these metrics, higher values indicate more widely differing compositions of gut microbiota. Substantial variation of gut microbiota within a subject was arbitrarily defined as JSD ≥ 0.4, which was higher than the median of inter-individual JSDs in this study.

### Estimation of the frequency of intake of fermented milk products

The intake frequency of fermented milk products containing LcS was estimated by a modified self-administered questionnaire^59,60^, with pictures of a series of commercially available LcS-containing products, including “Yakult”, “Joie”, “Soful”, and “Pretio” (Yakult Honsha Co. Ltd., Minato, Tokyo, Japan), each of which contains 0.9–40 billion live LcS per bottle. Subjects were asked how many days per week they had ingested products of the type illustrated over 1-month, 5-year, and 10-year periods before the collection of fecal samples. The intake frequency of general fermented milk products, such as yogurt (including the above Yakult Honsha Co. Ltd. products), was also estimated by the same procedure. Subjects were classed as consuming a bottle of the product less than 3 days per week (designated as “ < 3 days/week”) or 3 days or more per week (designated as “ ≥ 3 days/week”), as categorized in our previous report^36^.

### Measurement of blood pressure, physical activity patterns and physical health

Blood pressure was measured after 5 min of seated rest, using an automatic sphygmomanometer (BP-103iII; Colin Medical Technology Co. Ltd., Komaki, Aichi, Japan). At least one further measurement was made after a further 5-min rest if the initial reading suggested that an individual had become hypertensive (or rarely, hypotensive). Physical activity patterns were measured for 24 h per day over a 1-month period, using a uniaxial acceleration sensor (Lifecorder; Suzuken Co. Ltd., Nagoya, Aichi, Japan), as described previously^32–35^. The average number of steps taken per day and the daily cumulative duration of moderate-intensity exercise [activity demanding an energy expenditure greater than 3 metabolic equivalents (METs)] were calculated for each subject. Preferred and maximal walking speeds were determined over a 5-m distance, using a stopwatch (SVAE101; Seiko Corp., Minato, Tokyo, Japan), as described previously^61^. Subjects completed two trials to determine each of comfortable and maximal walking speeds; the averaged and the higher velocities were each recorded for each of the two measurements. Peak handgrip force was assessed for the dominant hand, using a Smedley dynamometer (ES-100; Evernew Co. Ltd., Koto, Tokyo, Japan). Two trials were performed, and the larger of the two readings was noted. Quantitative ultrasound measurements of osteosonic index for the calcaneus were made using an Achilles ultrasonic bone densitometer (AOS-100; Aloka Co. Ltd., Mitaka, Tokyo, Japan), as described previously^62^.

### Assessment of anthropometric characteristics, blood profiles and medical history

The physical characteristics of subjects (age, sex, height, body mass, and body mass index) were determined by standard anthropometric techniques^63^. Biochemical profiles (triglyceride, high-density lipoprotein cholesterol, low-density lipoprotein cholesterol, glycosylated hemoglobin A_1c_, blood sugar, glutamic oxaloacetic transaminase, glutamic pyruvic transaminase, γ-glutamyl transpeptidase, albumin, creatinine, uric acid concentrations, and the estimated glomerular filtration rate) were also measured by standard methods (Health Sciences Research Institute Inc., Yokohama, Kanagawa, Japan). The medical history was obtained from the doctor’s records.

### Investigation of nutrient intake

The nutritional status of the subjects was evaluated by a certified nutritionist over a 1-week period, using Version 3.5 of the Food Frequency Questionnaire Based on Food Groups^64^ (FFQg; Kenpakusha Co. Ltd., Bunkyo, Tokyo, Japan), which is a 20-item questionnaire regarding the consumption of items from 29 food groups and 10 methods of food preparation. On the basis of responses to this questionnaire, the daily intake of energy, nutrients, and food groups was estimated for the 1- to 2-month period prior to the fecal sampling. The estimated nutrients included protein, lipid, carbohydrate, dietary fiber, saturated fatty acids, monounsaturated fatty acids, polyunsaturated fatty acids, cholesterol, sodium, potassium, calcium, magnesium, iron, and vitamin C.

### Determination of defecation and medication status

The frequency of defecation was estimated by a self-administered questionnaire. Subjects were asked how many days they had defecated over a 1-week period before the fecal sampling. The consistency of the feces was estimated using the Bristol Stool Form Scale^65^ (BSFS), a diagnostic tool designed to classify the shape and type of human feces into seven distinct categories: separate hard lumps, like nuts (= 1); sausage-shaped, but lumpy (= 2); like a sausage, but with cracks on its surface (= 3); like a sausage or snake, smooth and soft (= 4); soft blobs with clear-cut edges (= 5); fluffy pieces with ragged edges, a mushy stool (= 6); and watery, no solid pieces, entirely liquid (= 7). The medication status was estimated using a self-administered questionnaire. Subjects filled out all the names of antibiotics and gastric acid lowering medicines that they had taken over a 1-week period before the fecal sampling.

### Statistical analyses

Version 3.3 of the R software (https://cran.r-project.org/) was used throughout^58^. Steel’s tests assessed differences between the values of intra-individual JSD and inter-individual JSD. Analytical subjects were divided into two groups, based on the intra-individual JSD (< 0.4 and ≥ 0.4) or based on the intake frequency of fermented milk products (< 3 and ≥ 3 days/week) over 1-month, 5-year, and 10-year periods. Analyses of covariance assessed independent differences between groups with respect to anthropometry, physical activity, defecation, physical health, fermented milk products consumption, nutrition, and/or blood characteristics, after controlling data for age and/or sex or with respect to intra-individual JSD and BCD, after controlling for age, sex, body mass index, smoking status and alcohol consumption. Chi-square tests assessed differences between groups in male/female ratio and the percentages of current smokers, current alcohol consumers, and medicine users. Fisher’s exact tests assessed differences between groups in terms of the prevalence of diseases. Permutational multivariat analyses of variance^66^ with 10,000 permutations assessed differences between groups with respect to gut microbiota at the family level in the first and second year, using JSD metrics as dissimilarity indices^67^. Spearman’s rank correlation analyses assessed associations between JSD_1st–2nd_ and JSD_2nd–3rd_. Fisher’s exact tests assessed differences between groups in terms of the incidence of substantial variation of gut microbiota (JSD ≥ 0.4). Levene’s tests assessed differences between groups in terms of the dispersions of changes in the relative abundance of gut microbiota families. All statistical contrasts were made at the 0.05 level of significance.

### Ethics approval

The protocol was approved and the study was carried out in accordance with the recommendations of the ethics review committee of the Tokyo Metropolitan Institute of Gerontology with written informed consent provided by all subjects in accordance with the Declaration of Helsinki.

## Supplementary Information

Supplementary Information 1.

## Data Availability

Gut 16S rRNA gene amplicon sequencing data have been deposited in the NCBI’s Sequence Read Archive (SRA) under the BioProject accession code PRJNA686801. The authors declare that all other data supporting the findings of this study are available in this article and its Supplementary File, or from the corresponding author upon request.
