# Author Correction: Yearly changes in the composition of gut microbiota in the elderly, and the effect of lactobacilli intake on these changes

**DOI:** 10.1038/s41598-021-95564-9

**Published:** 2021-08-04

**Authors:** Ryuta Amamoto, Kazuhito Shimamoto, Sungjin Park, Hoshitaka Matsumoto, Kensuke Shimizu, Miyuki Katto, Hirokazu Tsuji, Satoshi Matsubara, Roy J. Shephard, Yukitoshi Aoyagi

**Affiliations:** 1 Food Research Department, Yakult Central Institute, Kunitachi, Tokyo Japan; 2 Exercise Sciences Research Group, Tokyo Metropolitan Institute of Gerontology, Itabashi, Tokyo Japan; 3 Microbiological Research Department, Yakult Central Institute, Kunitachi, Tokyo Japan; 4 Basic Research Department, Yakult Central Institute, Kunitachi, Tokyo Japan; 5 Faculty of Kinesiology and Physical Education, University of Toronto, Toronto, ON Canada

Correction to: *Scientific Reports* 10.1038/s41598-021-91917-6, published online 17 June 2021

The original version of this Article contained a repeated error in the Abstract, Introduction, the legends of Figure 4 and 5, the legend of Table 2, and in the Supplementary Information file, where

“*Lactocaseibacillus paracasei*”.

now reads:

“*Lacticaseibacillus paracasei*”.

The original Article and accompanying Supplementary Information file have been corrected.

