# Supplementary material for: Yearly changes in the composition of gut microbiota in the elderly, and the effect of lactobacilli intake on these changes
Source: Sci Rep. 2021 Jun 17;11:12765. doi: 10.1038/s41598-021-91917-6 (PMC8211673; doi:10.1038/s41598-021-91917-6)
Supplement: Supplementary file 1 — Supplementary Information 1. [file 41598_2021_91917_MOESM1_ESM.docx]

**Supplementary Figures and Tables**

**Yearly changes in the composition of gut microbiota in the elderly, and the effect of lactobacilli intake on these changes**

Ryuta Amamoto^1^, Kazuhito Shimamoto^1^, Sungjin Park^2^, Hoshitaka Matsumoto^3^, Kensuke Shimizu^3^, Miyuki Katto^3^, Hirokazu Tsuji^4^, Satoshi Matsubara^1^, Roy J. Shephard^5^, Yukitoshi Aoyagi^2^*

1 Food Research Department, Yakult Central Institute, Kunitachi, Tokyo, Japan

2 Exercise Sciences Research Group, Tokyo Metropolitan Institute of Gerontology, Itabashi, Tokyo, Japan

3 Microbiological Research Department, Yakult Central Institute, Kunitachi, Tokyo, Japan

4 Basic Research Department, Yakult Central Institute, Kunitachi, Tokyo, Japan

5 Faculty of Kinesiology and Physical Education, University of Toronto, Toronto, ON, Canada

**^*^** **Correspondence:**

Yukitoshi Aoyagi, Ph.D.

[aoyagi@tmig.or.jp](mailto:aoyagi@tmig.or.jp)


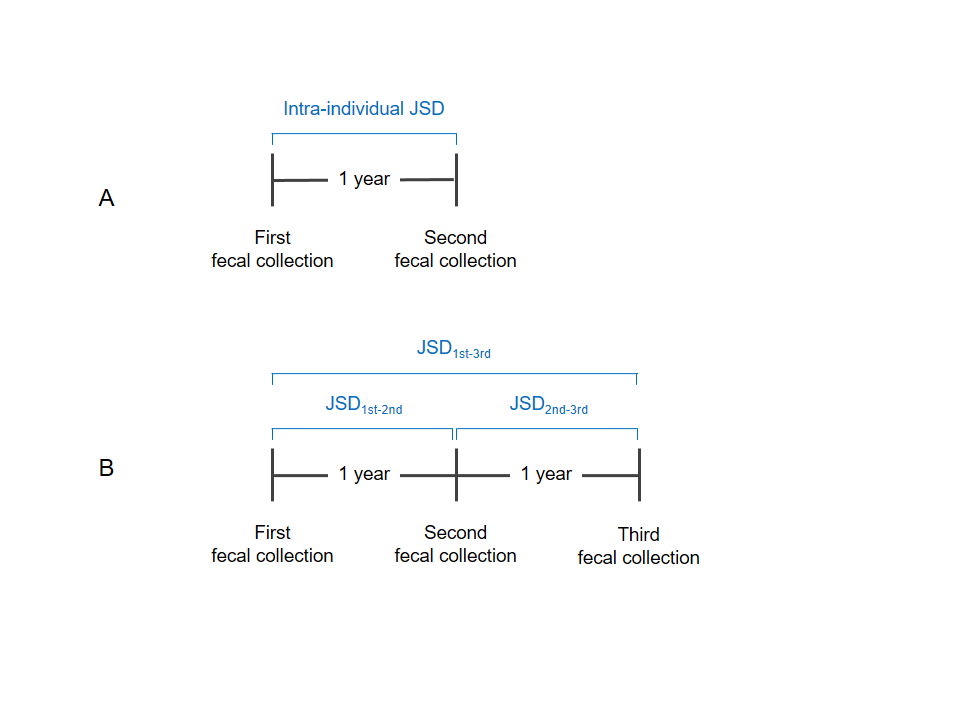


Supplementary Figure S1. Timeline of the fecal collection and the definition of each Jensen-Shannon distance (JSD). (A) Subjects who provided their fecal samples annually for 2 consecutive years (n = 218). (B) Subjects who provided their fecal samples annually for 3 consecutive years (n = 135).


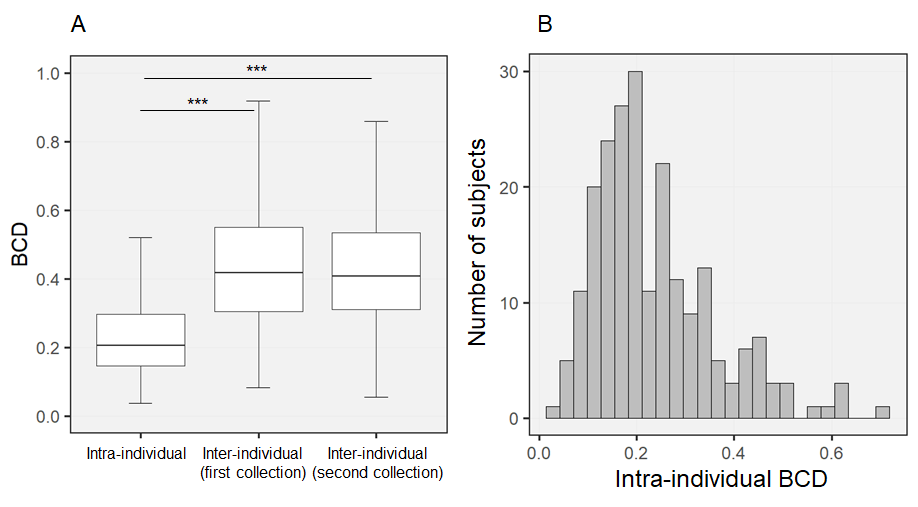


Supplementary Figure S2. Intra-individual variations and inter-individual differences in gut microbial composition, quantified by Bray-Curtis dissimilarity (BCD). (A) Comparison of BCD values between intra-individuals and inter-individuals by Steel’s tests. *** *P* < 0.001. (B) Distribution of intra-individual variations among the 218 subjects.


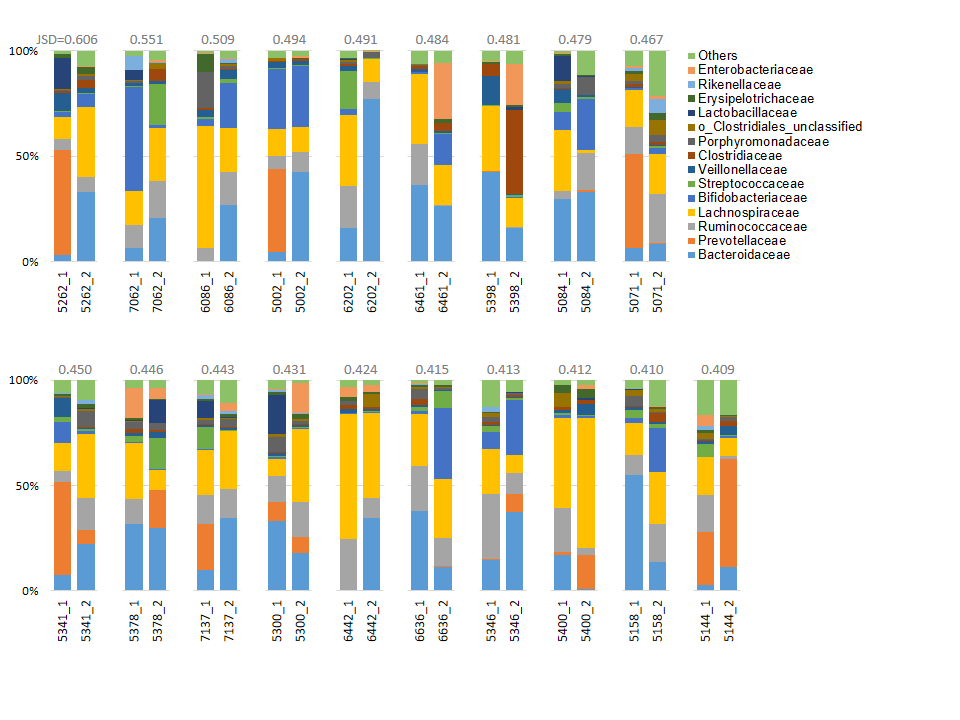


Supplementary Figure S3. Changes of the predominant gut microbial composition at the family level between the first and second year in 19 subjects with a Jensen-Shannon distance (JSD) ≥ 0.4.


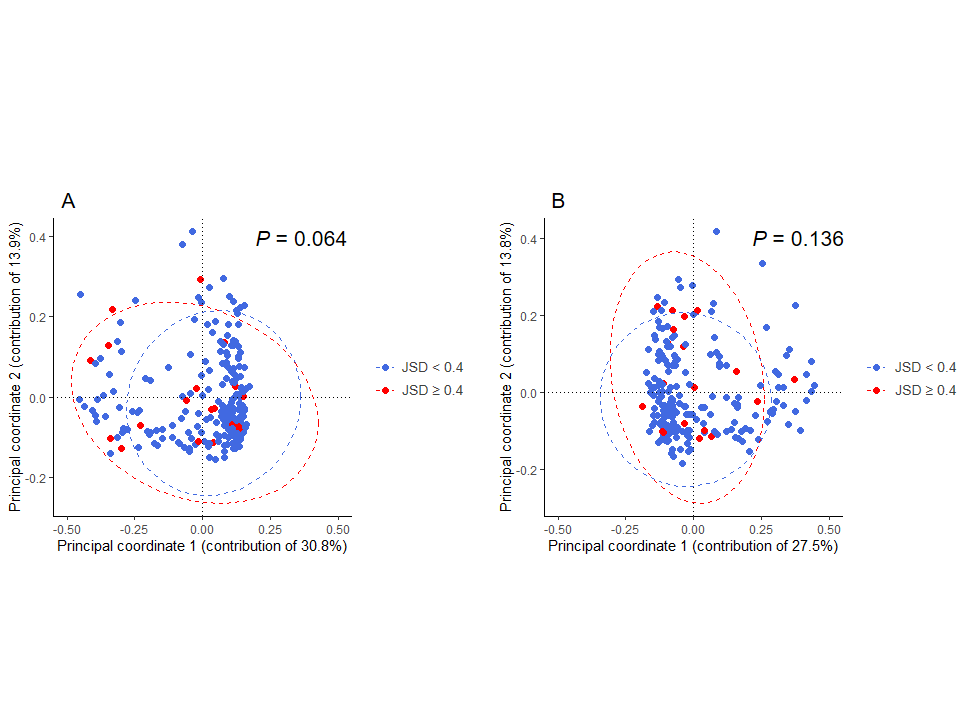


Supplementary Figure S4. Principal coordinate analysis plots with respect to associations between intra-individual Jensen-Shannon distance (JSD) and family-level gut microbiota at the time of fecal collection in the first (A) and second (B) year. Differences in the gut microbiota between subjects with JSDs < 0.4 (n=199) and ≥ 0.4 (n=19) were assessed by permutational multivariat analyses of variance with 10,000 permutations, using JSD metrics as dissimilarity indices.


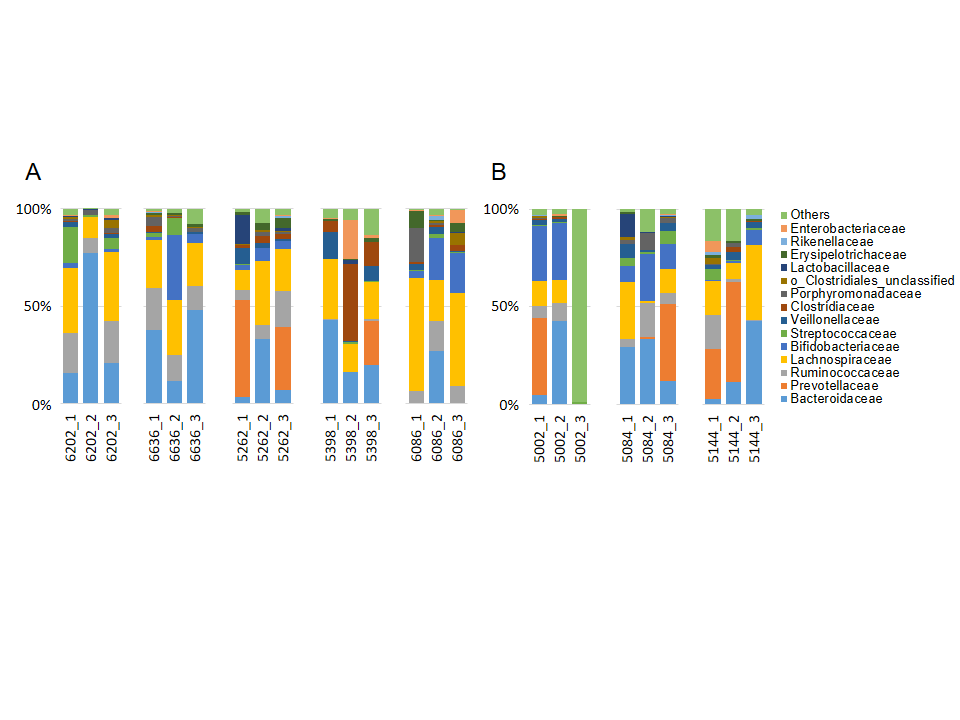


Supplementary Figure S5. Changes of the predominant gut microbial composition at the family level in 3 years among subjects with both JSD_1st-2nd_ and JSD_2nd-3rd_ ≥ 0.4. (A) Subjects whose gut microbiota composition returned to the initial state after a substantial change. (B) Subjects with substantial changes in gut microbiota in 3 years.


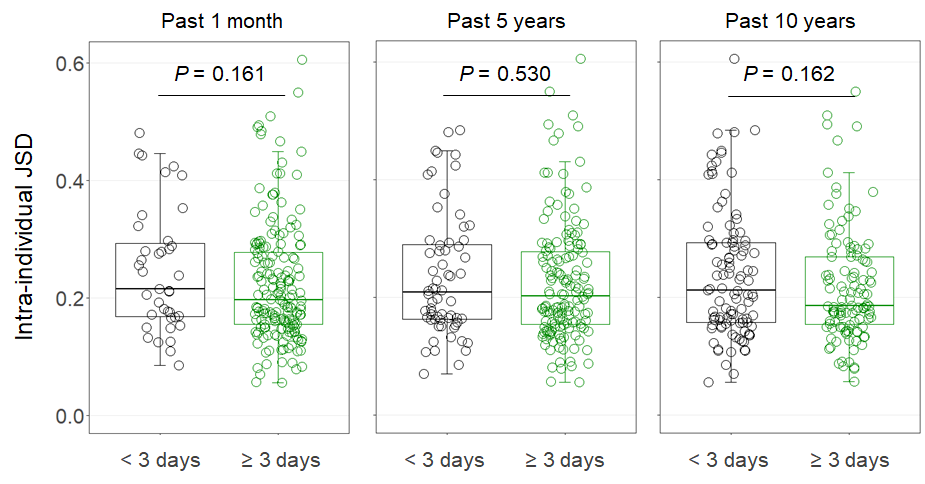


Supplementary Figure S6. Intra-individual Jensen-Shannon distance (JSD) in subjects consuming overall fermented milk products < 3 or ≥ 3 days/week during the past 1-month, 5-year and 10-year periods. Independent differences in the JSD between groups were assessed by analyses of covariance, after adjusting data for age, sex, body mass index, smoking status, and alcohol intake.


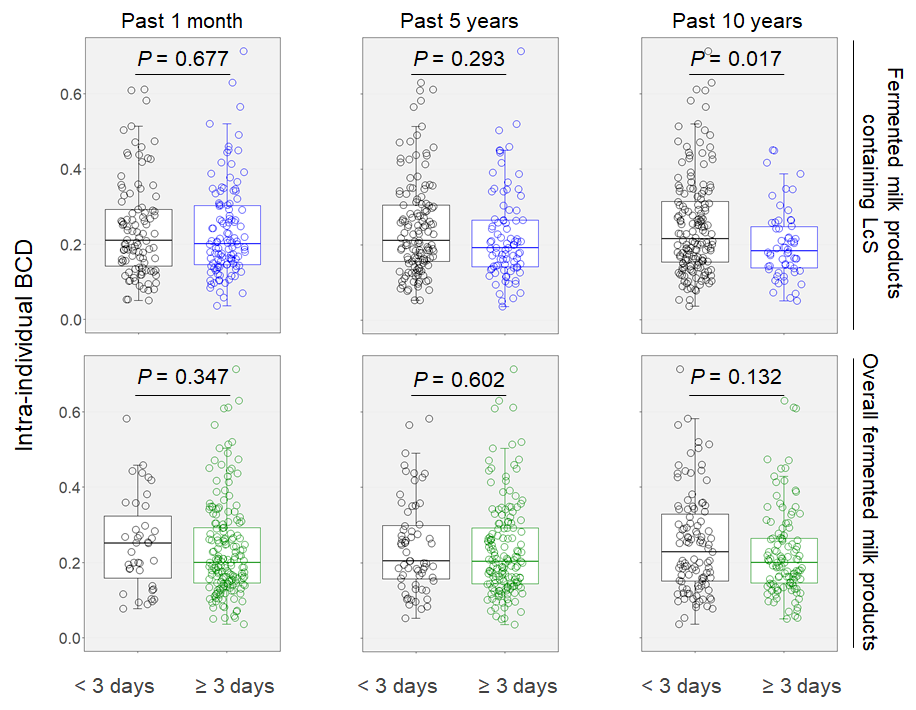


Supplementary Figure S7. Intra-individual Bray-Curtis dissimilarity (BCD) in subjects consuming fermented milk products < 3 or ≥ 3 days/week during the past 1-month, 5-year and 10-year periods. Upper panel, fermented milk products containing *Lacticaseibacillus paracasei* strain Shirota (LcS). Lower panel, overall fermented milk products. Independent differences in the BCD between groups were assessed by analyses of covariance, after adjusting data for age, sex, body mass index, smoking status, and alcohol intake.

Supplementary Table S1. Prevalence of disease in subjects with Jensen-Shannon distance (JSD) < 0.4 or ≥ 0.4

|  | < 0.4 | | | |  | ≥ 0.4 | | | |
| --- | --- | --- | --- | --- | --- | --- | --- | --- | --- |
|  | Case | / | n | Prevalence (%) |  | Case | / | n | Prevalence (%) |
| Hypertension | 92 | / | 199 | (46.2) |  | 10 | / | 19 | (52.6) |
| Hyperlipidemia^1^ | 50 | / | 199 | (25.1) |  | 3 | / | 19 | (15.8) |
| Anemia | 29 | / | 199 | (14.6) |  | 4 | / | 19 | (21.1) |
| Diabetes | 21 | / | 199 | (10.6) |  | 0 | / | 19 | (0.0) |
| Osteoporosis | 17 | / | 199 | (8.5) |  | 2 | / | 19 | (10.5) |
| Prostatic hypertrophy^2^ | 16 | / | 91 | (17.6) |  | 2 | / | 8 | (25.0) |
| Cataract | 15 | / | 199 | (7.5) |  | 1 | / | 19 | (5.3) |
| Angina | 11 | / | 199 | (5.5) |  | 2 | / | 19 | (10.5) |
| Glaucoma | 11 | / | 199 | (5.5) |  | 1 | / | 19 | (5.3) |
| Stroke^3^ | 11 | / | 199 | (5.5) |  | 0 | / | 19 | (0.0) |
| Arrhythmia | 8 | / | 199 | (4.0) |  | 2 | / | 19 | (10.5) |

^1^ Hyperlipidemia includes hypercholesterolemia, hypertriglyceridemia and dyslipidemia.

^2^ Prostatic hypertrophy is calculated for males only.

^3^ Stroke includes cerebral infarction, cerebral hemorrhage and subarachnoid hemorrhage.

Independent differences in the prevalence of disease between JSD < 0.4 and ≥ 0.4 were assessed by Fisher’s exact tests. There were no statistically significant differences in the prevalence of disease between the two groups. Diseases with total number of cases ≥ 10 were shown.

Supplementary Table S2. Changes in the medical history, medication status and lifestyle of subjects who experienced the major variation in gut microbiota (Jensen-Shannon distance ≥ 0.4) during a year.

| ID | Age | Sex | Development of  new diseases | Changes of medication status | |  | Changes of lifestyle |
| --- | --- | --- | --- | --- | --- | --- | --- |
|  |  |  |  | Antibiotics | Gastric acid lowering medicines |  |  |
| 5262 | 69 | Male | No | No | No |  | No |
| 7062 | 73 | Female | No | No | No |  | No |
| 6086 | 67 | Female | No | No | No |  | No |
| 5002 | 75 | Female | Ischemic enteritis | No | No |  | No |
| 6202 | 85 | Female | No | Yes | No |  | Physical activity decreased |
| 6461 | 72 | Male | Choledocholithiasis | No | No |  | No |
| 5398 | 78 | Male | No | No | No |  | No |
| 5084 | 85 | Male | No | Yes | No |  | No |
| 5071 | 68 | Female | No | No | No |  | Alcohol intake began |
| 5341 | 85 | Male | No | No | No |  | Physical activity decreased |
| 5378 | 78 | Male | No | No | No |  | No |
| 7137 | 68 | Male | No | No | No |  | No |
| 5300 | 81 | Female | No | No | Yes |  | No |
| 6442 | 76 | Female | No | No | No |  | No |
| 6636 | 77 | Male | No | No | No |  | No |
| 5346 | 72 | Female | No | No | No |  | Physical activity increased |
| 5400 | 69 | Female | No | No | No |  | No |
| 5158 | 66 | Female | No | No | No |  | No |
| 5144 | 68 | Female | No | No | No |  | No |

Changes of lifestyle indicate changes in smoking status, alcohol intake, and daily physical activity (step count and duration of moderate-intensity activity) by 30% or more over a 1-year period.

Supplementary Table S3. Frequency distribution of consumption of fermented milk products containing *Lacticaseibacillus paracasei* strain Shirota (LcS) and overall fermented milk products in sample of 218 elderly Japanese.

|  | Intake frequency (days/week) | | | | | | | |
| --- | --- | --- | --- | --- | --- | --- | --- | --- |
|  | 0 | 1 | 2 | 3 | 4 | 5 | 6 | 7 |
| LcS fermented milk products |  |  |  |  |  |  |  |  |
| Past 1 month | 70 | 18 | 14 | 19 | 15 | 9 | 14 | 59 |
| Past 5 years | 76 | 33 | 24 | 26 | 15 | 14 | 5 | 25 |
| Past 10 years | 96 | 31 | 33 | 22 | 9 | 6 | 2 | 19 |
| Overall fermented milk products |  |  |  |  |  |  |  |  |
| Past 1 month | 17 | 10 | 12 | 16 | 15 | 16 | 17 | 115 |
| Past 5 years | 30 | 15 | 19 | 32 | 18 | 21 | 12 | 71 |
| Past 10 years | 42 | 22 | 39 | 27 | 18 | 13 | 4 | 53 |
